# Rotational excitation of C_2_H^−^ anion in collision with H_2_†

**DOI:** 10.1039/d1ra00519g

**Published:** 2021-04-13

**Authors:** Insaf Toumi, Ounaies Yazidi, Faouzi Najar

**Affiliations:** Laboratoire de Spectroscopie Atomique, Moléculaire et Application, Faculté des Sciences, Université Tunis El Manar Tunis 2092 Tunisia; Institut Préparatoire aux Etudes d'Ingénieurs de Tunis El Manar, Université de Tunis El Manar Tunis 2092 Tunisia; Institut Préparatoire aux Etudes d'Ingénieurs de Tunis, Université de Tunis Tunis 1007 Tunisia

## Abstract

The discovery of anions in the interstellar medium has shown that they are very reactive species. This gave them great importance in the modeling of the chemical and astrophysical evolution of the interstellar medium. The detection of the first anion C_6_H^−^ followed by the other anions C_4_H^−^, C_8_H^−^ and CN^−^ in the interstellar medium has encouraged research on other detectable anions. The C_2_H^−^ anion was observed for the first time in the circumstellar envelope of IRC+10216 and in TMC-1. In these cold and low-density regions, precise modeling of the chemical and physical conditions of the observed emission lines requires knowledge of the radiative and collisional excitation rates. We present here the first new two-dimensional Potential Energy Surface (PES) for C_2_H–H_2_ interaction. Rotational excitation of the anion by collision with *para*-H_2_(*j*_H_2__ = 0) is investigated. The PES is obtained in the super-molecular approach based on a single and double excitation coupled cluster method with perturbative contributions from triple excitations (CCSD(T)). In all our calculations, all atoms were described using the augmented correlation-consistent triple zeta (aug-cc-pVTZ) basis sets and bond f unctions. Fully-quantum close-coupling calculations of inelastic integral cross sections are done on a grid of collision energies large enough to ensure converged state-to-state rate coefficients for the 16 first rotational levels of C_2_H^−^ and for temperatures ranging from 5 to 120 K. For this collisional system, rate coefficients exhibit a strong propensity in favor of even Δ*j* transitions.

## Abstract 2

The discovery of anions in the interstellar medium has shown that they are very reactive species.

## Graphical abstract

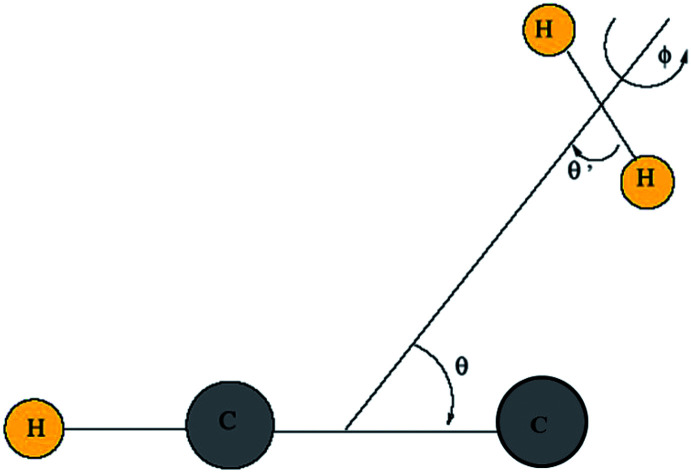

## 1 Introduction

In the last two decades, anions have been intensively studied. These studies were framed by observations in the laboratory^1–3^ and some astrophysical observations.^2,4–6^ Among these species, mention is made of carbon chain anions C_2*n*_H^−^.^6^ The recent discovery of carbon chain anions C_2*n*_H^−^ in interstellar and circumstellar media has been investigated by many theoretical and experimental works on these species.^7,8^ Their structures as well as the importance of their role in the interstellar chemistry and in gas phase ion–molecule reactions are the object of many recent studies.^9–11^ Although the existence of anions in astrophysical sources was first predicted theoretically and early considered in chemical models,^12,13^ the first negative hydrocarbon C_6_H^−^ was detected in 2006 (ref. 2) solving the problem of the unidentified lines discovered by Kawaguchi *et al.*^14^ The C_6_H^−^ identification was followed by the detection of other negatively charged species like C_4_H^−^, C_8_H^−^, C_3_N^−^, C_5_N^−^ and CN^−^.^15–23^ Many of these species were first detected in IRC+10216.^2,15,18^ Hydrocarbon anions were also discovered later in other molecular clouds.^17^ In this set C_2_H^−^ present a capital importance.

Several observations in the laboratories have justified the detection of the C_2_H^−^ anion. This experimental studies^24^ interest of the C_2_H^−^ anion comes from the fact that the C_2_H^−^ anion is the shortest in the sequence of carbon chain anions with a closed-shell electronic ground state, and the fact that gives the rest frequencies required for a radio astronomical search for this polar, astronomically plausible molecule.

From astrophysical observations, in 2007 J. Cernicharo *et al.*^15,25^ detected the 1–0 transition of the C_2_H^−^ anion and calculated the abundance ratio C_2_H/C_2_H^−^ = 12.5. Cernicharo *et al.* suggest that the 3–2 transition is strong enough to be detected. Later in 2010, the observations of M. Agùndez *et al.*^26^ found difficulties in detecting the 1–0 and 2–0 transitions of the anion. These difficulties are probably due to the very reactive natures of the C_2_H^−^ anion.

We report in this work a first collisional study of C_2_H^−^ with *para*-H_2_(*j*_H_2__ = 0) at low temperatures trying to understand the particular behavior of negatively charged species during collisions and how they compare with neutral forms^27,47^ and the C_2_H^−^ anion in collision with He.^48^ In the next section, we will present details of the *ab initio* calculations of the C_2_H–*para*-H_2_ potential Energy Surface (PES).In Section 3, some theoretical aspects of the scattering calculations are given, state-to-state collisional cross sections will be reported, the rate coefficients and the propensity rules will be showcased. In Section 4, comparative study between the rate coefficients of C_2_H^−^–*para*-H_2_ and C_2_H–He will be displayed. Concluding remarks are in Section 5.

## 2 Potential Energy Surface

This work primarily focuses on the rotational excitation of C_2_H^−^ with *para*-H_2_ at low temperature; both monomers were considered as rigid species to deal with a reduced number of degrees of freedom. Accordingly, in our calculations, is considered to be linear, and both bond lengths are set at the CCSD/AV5Z theoretical values of *r*_C–C_ = 1.250 Å and *r*_C–H_ = 1.070 Å.^28^ For H_2_, we used the experimental bond length *r*_H–H_ = 1.44876 bohr corresponding to the averaged value over the ground-state vibrational wave function for H_2_. The lowest bending mode of C_2_H^−^*ν*_1_ equal to 502.0 cm^−1^,^28^ is well above the energy of the highest C_2_H^−^ rotational level considered in this work (*j* = 16, see Table 1). In addition, from QCT studies of rotational excitation of H_2_O by H_2_, it was shown^29^ that the coupling between the rotational excitation and the bending may be neglected for temperatures up to 5000 K. Moreover, it was also shown by Faure *et al.*,^30^ in the particular case of the H_2_O–H_2_ system, that the 5D-PES calculated at the experimental ground vibrational state geometry and the full 9D-PES averaged over the ground vibrational state are very similar. These results suggest that the use of a rigid body 2D-PES is sufficient as long as bending and vibrational excitations are not taken into consideration.

**Table 1:** Rotational Energy for the first 28 Levels of C_2_H^−^ system

*j*	*ε*_*j*_ (cm^−1^)	*j*	*ε*_*j*_ (cm^−1^)
0	0.0000000		
1	2.7778579	15	333.1581888
2	8.3334960	16	377.5511276
3	16.6667590	17	424.7113668
4	27.7774142	18	474.6375867
5	41.6651509	19	527.3283902
6	58.3295811	20	582.7823022
7	77.7702390	21	640.9977702
8	99.9865812	22	701.9731640
9	124.9779866	23	765.7067757
10	152.7437566	24	832.1968200
11	183.2831149	25	901.4414338
12	216.5952076	26	973.4386763
13	252.6791032	27	1048.1865292
14	291.5337926	28	1125.6828966

**Fig. 1:**
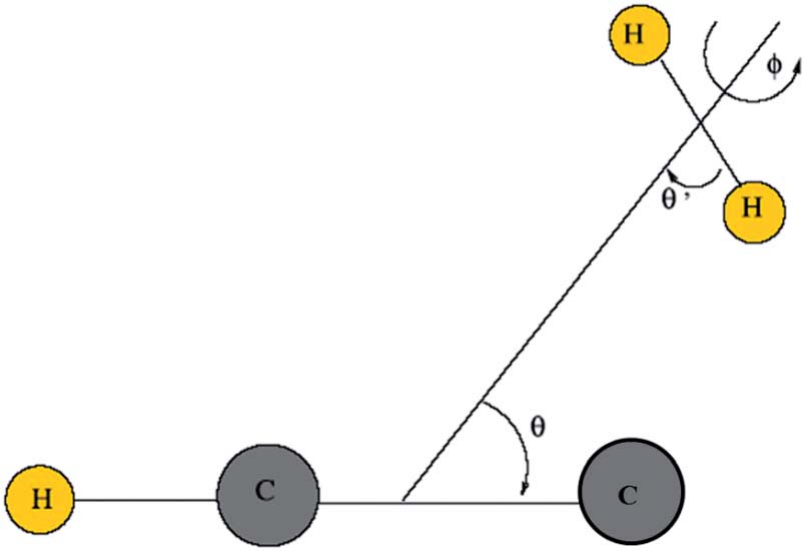
Definition of the body-fixed coordinate system for C_2_H^−^–H_2_.

Then, we define the body-fixed coordinate system in Fig. 1. The geometry of C_2_H^−^–H_2_ collisional system with *para*-H_2_ and C_2_H^−^ treated as rigid rotors is then characterized by three angles (*θ*, *θ*′, *ϕ*) and the distance *R* between the center of masses of H_2_ and C_2_H^−^ (see Fig. 1). The polar angles of the C_2_H^−^ and *para*-H_2_ molecules with respect to *R* are denoted *θ* and *θ*′ respectively, while *ϕ* denotes the dihedral angle, which is the relative polar angle between the C_2_H^−^ and *para*-H_2_ bonds. For the solution of the close-coupling scattering equations, it is most convenient to expand, at each value of *R*, the interaction potential *V*(*R*,*θ*,*θ*′,*ϕ*) in angular functions. For the scattering of two linear rigid rotors, we used.^31^1
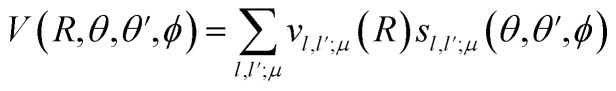
The basis functions *s*_*l*,*l*′;*μ*_(*θ*,*θ*′,*ϕ*) are products of normalized associated Legendre functions *P*_*lm*_:2
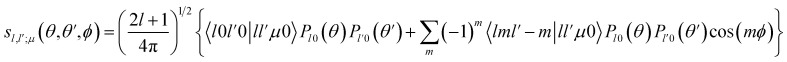
where 〈…|…〉 is a Clebsch–Gordan coefficient. The *P*_*lm*_ functions are related to spherical harmonics through *Y*_*lm*_(*θ*,*ϕ*) = *P*_*lm*_(*θ*)exp(*imϕ*). Here, *l* and *l*′ are associated with the rotational motion of C_2_H^−^ and *para*-H_2_ respectively. In eqn (1) the homonuclear symmetry of H_2_ forces the index *l*′ to be even. For collisions at low/moderate temperature, the probability of rotational excitation of H_2_ is low (the energy spacing between the *j*_H_2__ = 0 and *j*_H_2__ = 2 levels in *para*-H_2_ being 510 K) so we further restrict H_2_ to its lowest rotational level. In this case, only the leading term 
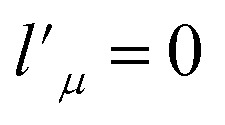
 needs to be retained in the expansion of the interaction potential given in eqn (1). The resulting expansion then can be simplified to3
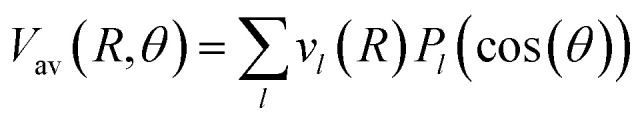
where the *V*_av_(*R*,*θ*) is obtained by an average over angular motion (*θ*′,*ϕ*) of the H_2_ molecule. We approximate the average by an equipoise averaging over three sets of (*θ*′,*ϕ*) angles for each calculated set of (*R*,*θ*). Those are 
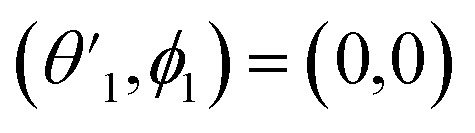
, 
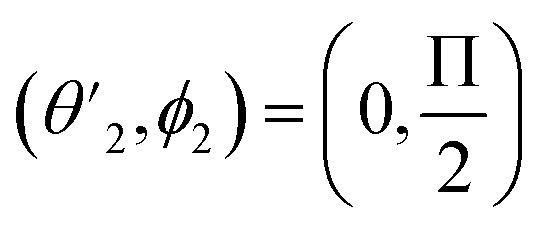
 and 
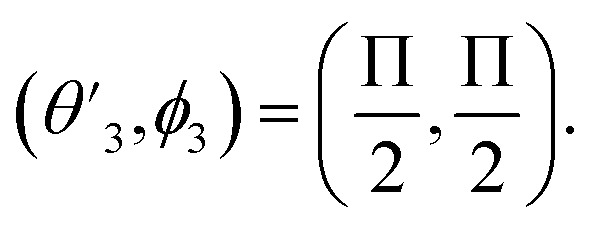
 Thus, the C_2_H^−^–*para*-H_2_(*j*_H_2__ = 0) is reduced to a two-dimensional *V*_av_ PES such as4
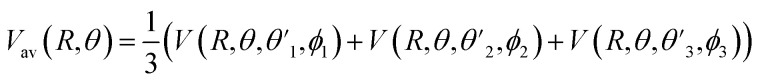


Such approximation has been shown to be reasonably accurate for relatively heavy molecule such as SiS,^32^ HCO^+^ (ref. 33) and C_2_.^34^ In the *C*_s_ point group, the ground electronic state of the C_2_H^−^–*para*-H_2_ van der Waals system is of *A*′ symmetry. The PES was calculated in the super-molecular approach based on a single and double excitation coupled cluster method with perturbative contributions from triple excitations (CCSD(T)).^35,36^ For the five atoms, we used the aug-cc-pVTZ basis set of Woon and Dunning.^37^ This basis set was further augmented by the additional 3s3p2d1f bond basis functions of Williams *et al.*^38^ and placed equidistant between the C_2_H^−^ and H_2_ centers of mass. At all geometries, the Boys and Bernardi^39^ counterpoise procedure was used to correct for basis set superposition error (BSSE). All calculations were carried out using the MOLPRO 2010 package.^40^

The radial scattering coordinate *R* was assigned 36 values ranging from 3.0 to 50.0 bohr, the *θ* grid ranged from 0° to 180° in steps of 15°. This resulted in a total of 1404 geometries computed for the C_2_H^−^–*para*-H_2_(*j*_H_2__ = 0) system.

An analytic representation of the present 2D PES suitable for dynamics calculations was obtained using the procedure described by eqn (12)–(16) of Werner *et al.*^41^ for the CN–He system. In order to perform the scattering calculations, this PES was expanded in terms of Legendre polynomials.

**Fig. 2:**
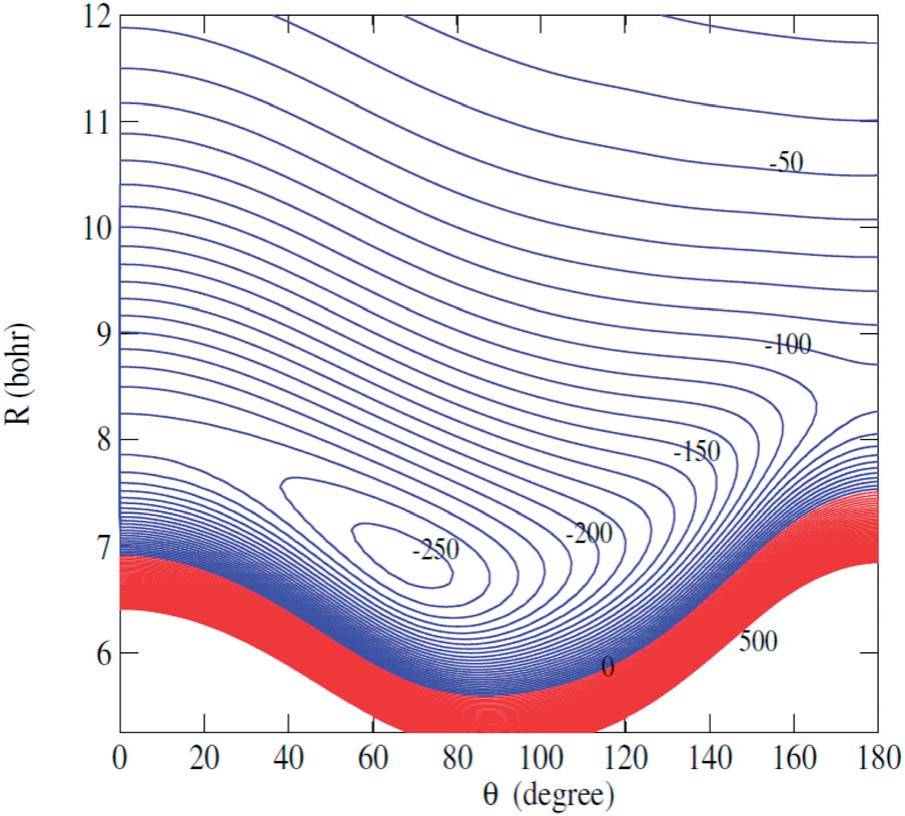
Contour plot of the C_2_H^−^–H_2_ PES as a function of *R* and *θ*.

Fig. 2 displays the contour plot of the 2D *V*(*R*,*θ*) PES. For this van der Waals system, the global minimum was found to be 250.6 cm^−1^ at *R* = 7.20 bohr and *θ* = 76.0 degree.

## 3 Dynamical calculation

The fitted C_2_H^−^–*para*-H_2_ 2D-PES was used to calculate state-to-state cross sections and rate coefficients. The full close coupling approach first introduced by Arthurs & Dalgarno^42^ was used for the calculations of the state-to-state cross sections between the 16 first rotational levels. The energies of the rotational levels were computed from the following C_2_H^−^ spectroscopic constants: *B*_0_ = 1.3889354 cm^−1^ and *D*_0_ = 3.2345 10^−6^ cm^−1^.^24^ The scattering calculations were carried out with the MOLSCAT code.^43^ Calculations were performed for energies ranging from 3.0 to 1200.0 cm^−1^. The integration parameters and the sise of the basis set were chosen to ensure convergence of the cross sections over this range. At the highest considered total energy (1200.0 cm^−1^), the C_2_H^−^ rotational basis included channels up to *j* = 28 to ensure convergence of the excitation cross sections for transitions including up to the *j* = 16 rotational level (see Tables 1 and 2).

**Table 2:** Convergence of cross-sections (in Å^2^) for some rotational transitions with respect to size of the basis set (*j*_max_) for total energy *E* = 1200 cm^−1^

	*j*_max_ = 27	*j*_max_ = 28	*j*_max_ = 29	*j*_max_ = 30	*j*_max_ = 32
*σ*_16–15_	15.316	15.322	15.322	15.322	15.322
*σ*_16–14_	6.666	6.665	6.665	6.665	6.663
*σ*_2–1_	2.7937	2.7941	2.7941	2.7941	2.7941

We carefully spanned the energy grid to take into account the presence of resonances. The energy steps are 0.1 cm^−1^ below 100.0 cm^−1^, 0.2 cm^−1^ from 100.0 to 250.0 cm^−1^, 1.0 cm^−1^ between 250.0 and 300.0, 5.0 cm^−1^ between 300.0 and 400.0 cm^−1^, 10.0 cm^−1^ between 400.0 and 500.0 cm^−1^, 25.0 cm^−1^ between 500.0 and 700.0 cm^−1^ and 50.0 cm^−1^ between 700.0 and 1200.0 cm^−1^.

### 3.1 Integral cross sections

**Fig. 3:**
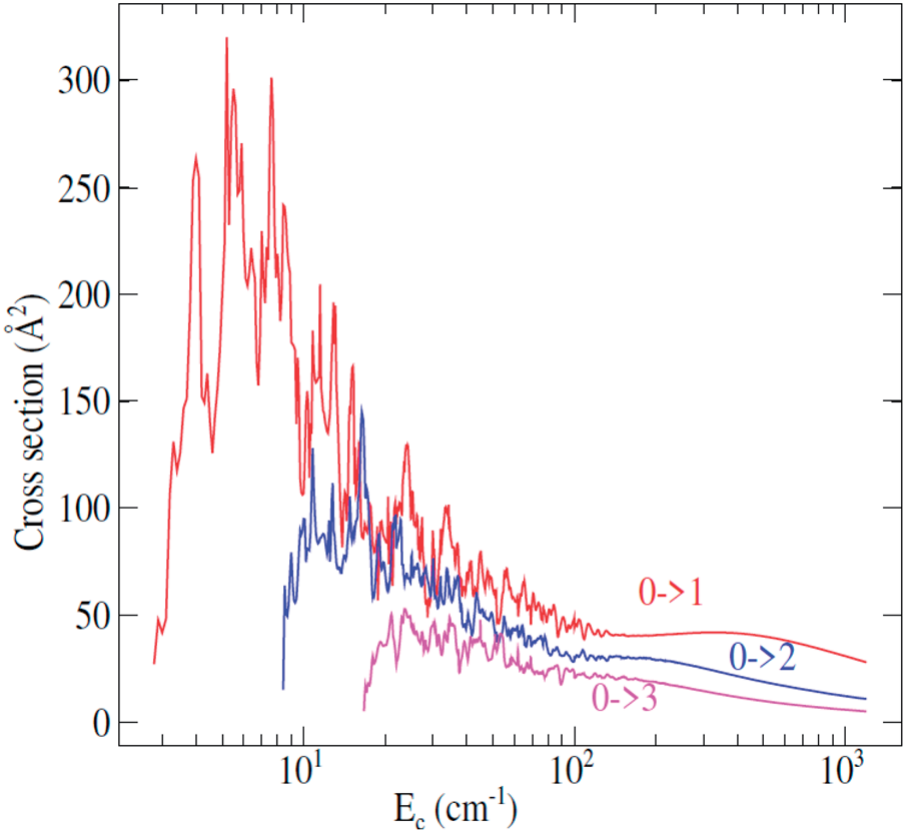
Rotational excitation *j* → *j*′ cross sections of C_2_H^−^ in collision with *para*-H_2_(*j*_H_2__ = 0) as a function of the relative kinetic energy.

Fully-quantum close-coupling calculations of integral cross sections were carried out for values of total energies ranging from 3.0 to 1200 cm^−1^. The variation of integral cross sections for a few selected *j* → *j*′ rotational transitions with collision energy is shown in Fig. 3. For collisional energies below 300.0 cm^−1^ many resonances occur. This is a consequence of the attractive potential well with a depth of 252.6 cm^−1^. At low energy, the molecule can be temporarily trapped in quasi-bound states,^44,45^ which arise both from tunneling through the centrifugal barrier (shape resonances) and from excitation of C_2_H^−^ to a higher rotational level which is energetically allowed in the well (Feshbach resonances). Some anisotropy appears around the well depth which may induce a different trend of the cross sections at lower energies.

### 3.2 Rate coefficients

**Fig. 4:**
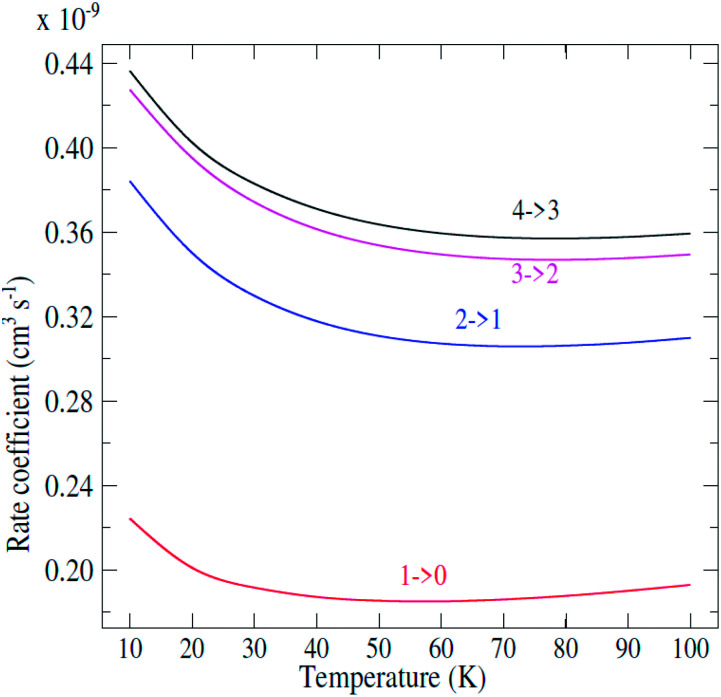
Temperature dependence of state-to-state rate coefficients.

**Fig. 5:**
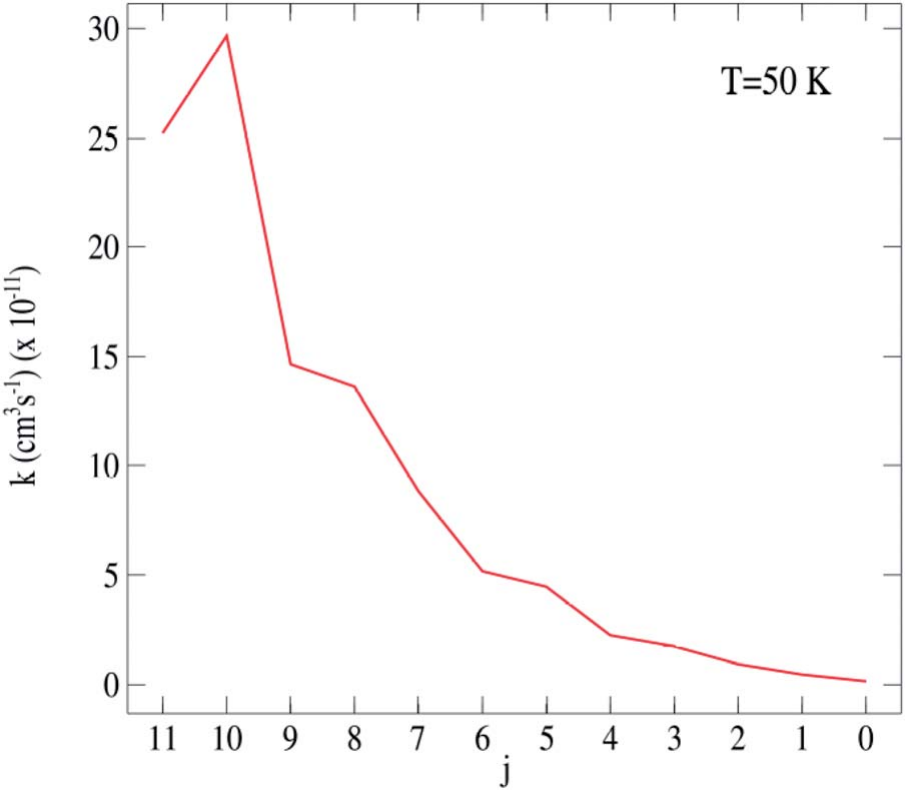
C_2_H^−^–*para*-H_2_(*j*_H_2__ = 0) excitation rate coefficients for the *j* → *j*′ = 12 transitions at 50 K.

From the calculated cross sections, one can obtain the corresponding state-to-state rate coefficients by Boltzmann averaging:5
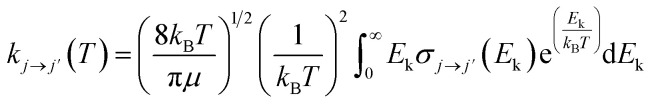
where *k*_B_ is the Boltzmann constant and *E*_k_ is the kinetic energy. The total energy range considered in this work allows us to determine rate coefficients for temperatures up to 120 K. The total energy range considered in this work allows us to determine rate coefficients for temperatures up to 120 K. The representative variation with temperature of de-excitation rate coefficients from an initial level *j* to a final level *j*′ is shown in Fig. 4. The rate coefficients obviously display the same propensity than the integral cross sections. Fig. 5 present for 50.0 K downward rotational rate coefficients of C_2_H^−^ (*j*^′^ = 12) level as a function of the final *j* level. This plot confirms the Δ*j* = 2 even propensity. The same behavior was found for the isoelectronic HCN molecule^46^ and for the neutral C_2_H^47^ in collision with *para*-H_2_(*j*_H_2__ = 0); and the C_2_H^−^ anion in collision with He.^48^

The complete set of de-excitation rate coefficients with *j*, *j*′ ≤ 16 will be available on-line from the BASECOL website (http://www.obspm.fr/basecol). Excitation rate coefficients can be easily obtained by detailed balance.

## 4 Comparison with C_2_H–He collisions

It is generally assumed that excitation rate coefficients associated with He as the collision partner can provide a first estimation for rate coefficients for collisions with *para*-H_2_(*j*_H_2__ = 0) just applying a scaling factor which is equal to the square root of the ratio of the reduced masses of the two systems. For C_2_H^−^, this scaling factor tends to 1.4. Since rotational rate coefficients have been calculated previously for C_2_H^−^ in collision with He,^48^ using the same methodology as the one employed in present work for C_2_H^−^–*para*-H_2_(*j*_H_2__ = 0), the comparison between the two sets of rate coefficients can be used to assess the validity of such approximation.

**Fig. 6:**
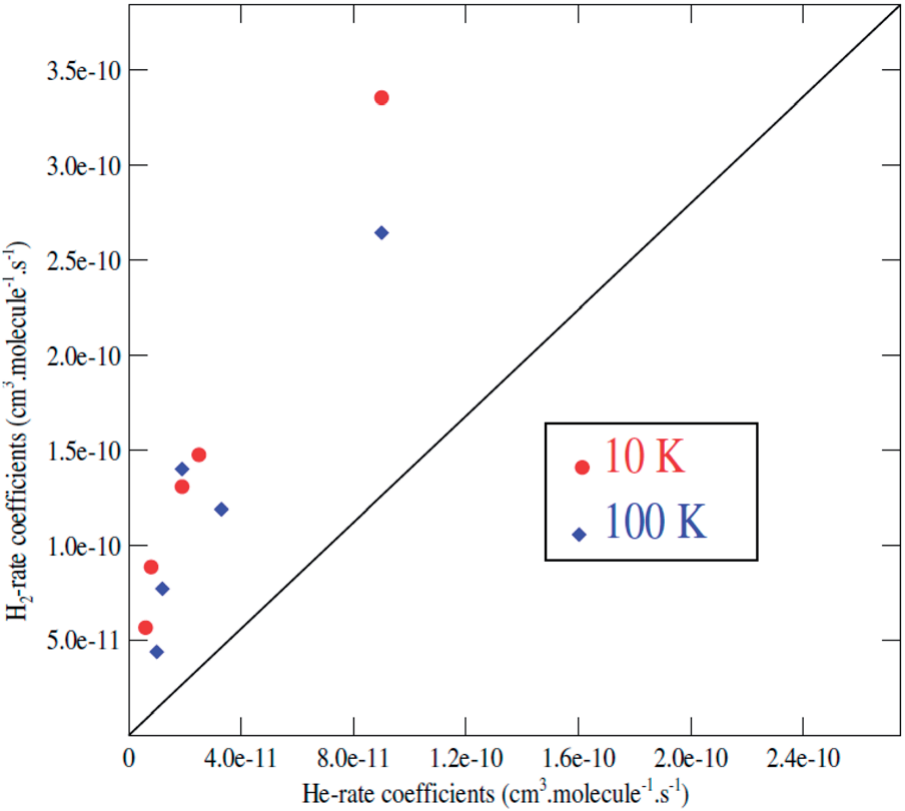
C_2_H^−^–*para*-H_2_(*j*_H_2__ = 0) rate coefficients as a function of the C_2_H–He rate coefficients for *j* → *j*′ transitions with *j* = 12 and *j*′ = 6, 7, 8, 9, 10, 11 at a temperature of 10 K (red circles) and 100 K (blue diamond); the black solid line corresponds to a ratio of 1.4.

We report in Fig. 6 a comparison between the rate coefficients of the present study and those reported by Dumouchel *et al.*^48^ for the selected transitions (see Fig. 4 in ref. 48). The red circles and the blue diamond's reveal the rate coefficients of C_2_H^−1^–*para*-H_2_(*j*_H_2__ = 0) *vs.* C_2_H^−^–He at kinetic temperatures *T* = 10.0 K and *T* = 100.0 K respectively. As shown above, the ratio between the rate coefficients clearly differs from the value of 1.4. These differences are due to the difference in interaction energies between the two systems. The ratios deviate clearly from 1.4 for all the transitions, by a factor varying from 3 to 11. The difference is more pronounced for the transitions with odd Δ*j*. This is due to the difference in the interaction potential which is more symmetrical (with respect to the *θ* ↔ π − *θ* transformation) for the C_2_H^−^–He system than for C_2_H^−1^–*para*-H_2_(*j*_H_2__ = 0). Then, the collision rates with corrected with the scale factor 1.4 do not always constitute a good approximation to model the rates with H_2_. This result is also found in the collisions with the neutral species C_2_H (see ref. 47).

## 5 Summary and conclusion

Quantum scattering calculations have been used to investigate energy transfer in collisions of C_2_H^−^ with *para*-H_2_(*j*_H_2__ = 0) molecule. The calculations are based on a new, highly correlated 2D PES calculated at the RCCSD(T) level using large AVTZ basis sets and a bend functions. Close coupling calculations were performed for collision energies ranging from 3.0 to 1200.0 cm^−1^. Rate coefficients for transitions involving the 16 first rotational levels of the C_2_H^−^ anion were determined for temperature ranging from 5 to 120.0 K. The rate coefficients show a strong propensity for even Δ*j*, mainly Δ*j* = 2. The present set of rate coefficients can serve the modelling of C_2_H^−^ emission lines in astrophysical environments. Finally, the resulting inelastic rate coefficients for collisions of C_2_H^−^ with *para*-H_2_ will help in constraining astrophysical anion chemistry and also in accurately model regions containing anions such as IRC+10216. We encourage astrophysicists to use these new values in their next detection attempts in the IRC+10216. Future work will deal with calculations of C_2_H^−^ in collision with both *para*- and *ortho*-H_2_ species using 4D PES approach in order to obtain rate coefficients for highly excited rotational states and higher temperatures. Detailed comparison with other systems, in particular carbon chain anions, will be studied in detail owing to its great interest for astrophysical modelling.

## Conflicts of interest

There are no conflicts to declare.

## Supplementary Material

RA-011-D1RA00519G-s001
