# Supplementary material for: Rotational excitation of C2H− anion in collision with H2
Source: RSC Adv. 2021 Apr 13;11(22):13579–84. doi: 10.1039/d1ra00519g (PMC8697610; doi:10.1039/d1ra00519g)
Supplement: RA-011-D1RA00519G-s001 [file RA-011-D1RA00519G-s001.pdf]

Electronic Supplementary Material (ESI) for RSC Advances.
This journal is © The Royal Society of Chemistry 2021

Rotational excitation of C_2H^- anion in collision with H_2

Insaf Toumi[a], Ounaies Yazidi[a,b], Faouzi Najar[a,c]

[a]Laboratoire de Spectroscopie Atomique, Moléculaire et Application, Faculté des Sciences, Université Tunis el Manar, Tunis 2092, Tunisia
[b]Institut Préparatoire aux Etudes d'Ingénieurs de Tunis El Manar, Université de Tunis El Manar, Tunis 2092, Tunisia
[c]Institut Préparatoire aux Etudes d'Ingénieurs de Tunis, Université de Tunis, Tunis 1007, Tunisia

(Supplementary material)

R	l	Vl(R)
5.250	0	1637.7148
5.500	0	960.9608
5.750	0	510.7201
6.000	0	217.2720
6.250	0	31.1449
6.500	0	-82.4633
6.750	0	-147.7505
7.000	0	-181.4013
7.250	0	-194.7615
7.500	0	-195.4780
7.750	0	-188.6271
8.000	0	-177.5847
8.250	0	-164.5130
8.500	0	-150.7592
8.750	0	-137.1591
9.000	0	-124.1979
9.250	0	-112.1506
9.500	0	-101.0735
9.750	0	-91.0283
10.000	0	-81.9974
10.250	0	-73.8962
10.500	0	-66.6560
10.750	0	-60.2111
11.000	0	-54.4750
11.250	0	-49.3616
11.500	0	-44.8051
11.750	0	-40.7450
12.000	0	-37.1206
12.250	0	-33.8753
12.500	0	-30.9681
12.750	0	-28.3623
13.000	0	-26.0209
13.250	0	-23.9097
13.500	0	-22.0057
13.750	0	-20.2885
14.000	0	-18.7379
14.250	0	-17.3344

14.500	0	-16.0622
14.750	0	-14.9065
15.000	0	-13.8524
15.250	0	-12.8864
15.500	0	-12.0013
15.750	0	-11.1916
16.000	0	-10.4513
16.250	0	-9.7750
16.500	0	-9.1569
16.750	0	-8.5914
17.000	0	-8.0726
17.250	0	-7.5950
17.500	0	-7.1529
17.750	0	-6.7412
18.000	0	-6.3576
18.250	0	-6.0003
18.500	0	-5.6677
18.750	0	-5.3579
19.000	0	-5.0693
19.250	0	-4.8002
19.500	0	-4.5488
19.750	0	-4.3134
20.000	0	-4.0923
20.250	0	-3.8840
20.500	0	-3.6877
20.750	0	-3.5030
21.000	0	-3.3294
21.250	0	-3.1664
21.500	0	-3.0134
21.750	0	-2.8701
22.000	0	-2.7358
22.250	0	-2.6100
22.500	0	-2.4924
22.750	0	-2.3824
23.000	0	-2.2794
23.250	0	-2.1830
23.500	0	-2.0928
23.750	0	-2.0081
24.000	0	-1.9285
24.250	0	-1.8535
24.500	0	-1.7825
24.750	0	-1.7152
25.000	0	-1.6510
25.250	0	-1.5894
25.500	0	-1.5304
25.750	0	-1.4738
26.000	0	-1.4195
26.250	0	-1.3674

26.500	0	-1.3175
26.750	0	-1.2697
27.000	0	-1.2238
27.250	0	-1.1798
27.500	0	-1.1376
27.750	0	-1.0971
28.000	0	-1.0582
28.250	0	-1.0208
28.500	0	-.9849
28.750	0	-.9503
29.000	0	-.9169
29.250	0	-.8847
29.500	0	-.8536
29.750	0	-.8235
30.000	0	-.7942
5.250	1	-779.7907
5.500	1	-498.0121
5.750	1	-324.0153
6.000	1	-217.7170
6.250	1	-153.8710
6.500	1	-116.0636
6.750	1	-93.8000
7.000	1	-80.5296
7.250	1	-72.2087
7.500	1	-66.4713
7.750	1	-61.9422
8.000	1	-57.9270
8.250	1	-54.0891
8.500	1	-50.2858
8.750	1	-46.5748
9.000	1	-42.8864
9.250	1	-39.2867
9.500	1	-35.8258
9.750	1	-32.5355
10.000	1	-29.4950
10.250	1	-26.6725
10.500	1	-24.0791
10.750	1	-21.7359
11.000	1	-19.6208
11.250	1	-17.7048
11.500	1	-15.9725
11.750	1	-14.4120
12.000	1	-13.0112
12.250	1	-11.7579
12.500	1	-10.6388
12.750	1	-9.6404
13.000	1	-8.7490
13.250	1	-7.9518

13.500	1	-7.2385
13.750	1	-6.5995
14.000	1	-6.0253
14.250	1	-5.5070
14.500	1	-5.0387
14.750	1	-4.6152
15.000	1	-4.2312
15.250	1	-3.8820
15.500	1	-3.5647
15.750	1	-3.2773
16.000	1	-3.0172
16.250	1	-2.7823
16.500	1	-2.5703
16.750	1	-2.3788
17.000	1	-2.2056
17.250	1	-2.0484
17.500	1	-1.9049
17.750	1	-1.7730
18.000	1	-1.6519
18.250	1	-1.5406
18.500	1	-1.4384
18.750	1	-1.3446
19.000	1	-1.2583
19.250	1	-1.1790
19.500	1	-1.1056
19.750	1	-1.0376
20.000	1	-.9741
20.250	1	-.9145
20.500	1	-.8585
20.750	1	-.8060
21.000	1	-.7568
21.250	1	-.7108
21.500	1	-.6679
21.750	1	-.6278
22.000	1	-.5905
22.250	1	-.5558
22.500	1	-.5236
22.750	1	-.4937
23.000	1	-.4659
23.250	1	-.4402
23.500	1	-.4163
23.750	1	-.3942
24.000	1	-.3736
24.250	1	-.3545
24.500	1	-.3367
24.750	1	-.3200
25.000	1	-.3043
25.250	1	-.2895

25.500	1	-.2755
25.750	1	-.2623
26.000	1	-.2498
26.250	1	-.2381
26.500	1	-.2270
26.750	1	-.2166
27.000	1	-.2068
27.250	1	-.1975
27.500	1	-.1888
27.750	1	-.1806
28.000	1	-.1728
28.250	1	-.1655
28.500	1	-.1586
28.750	1	-.1520
29.000	1	-.1458
29.250	1	-.1398
29.500	1	-.1341
29.750	1	-.1287
30.000	1	-.1234
5.250	2	3949.8037
5.500	2	2639.3678
5.750	2	1742.6653
6.000	2	1134.2800
6.250	2	725.7263
6.500	2	454.3364
6.750	2	276.0965
7.000	2	160.5763
7.250	2	86.8758
7.500	2	40.7660
7.750	2	12.6507
8.000	2	-3.8073
8.250	2	-12.8947
8.500	2	-17.4532
8.750	2	-19.0598
9.000	2	-19.0478
9.250	2	-18.1189
9.500	2	-16.7936
9.750	2	-15.2402
10.000	2	-13.6032
10.250	2	-12.0222
10.500	2	-10.5656
10.750	2	-9.2730
11.000	2	-8.1371
11.250	2	-7.1399
11.500	2	-6.2656
11.750	2	-5.4992
12.000	2	-4.8255
12.250	2	-4.2310

12.500	2	-3.7087
12.750	2	-3.2527
13.000	2	-2.8575
13.250	2	-2.5171
13.500	2	-2.2238
13.750	2	-1.9696
14.000	2	-1.7463
14.250	2	-1.5471
14.500	2	-1.3690
14.750	2	-1.2104
15.000	2	-1.0694
15.250	2	-.9446
15.500	2	-.8345
15.750	2	-.7379
16.000	2	-.6537
16.250	2	-.5805
16.500	2	-.5171
16.750	2	-.4624
17.000	2	-.4151
17.250	2	-.3739
17.500	2	-.3377
17.750	2	-.3054
18.000	2	-.2765
18.250	2	-.2508
18.500	2	-.2280
18.750	2	-.2078
19.000	2	-.1900
19.250	2	-.1742
19.500	2	-.1602
19.750	2	-.1478
20.000	2	-.1366
20.250	2	-.1264
20.500	2	-.1172
20.750	2	-.1088
21.000	2	-.1012
21.250	2	-.0944
21.500	2	-.0884
21.750	2	-.0830
22.000	2	-.0782
22.250	2	-.0740
22.500	2	-.0703
22.750	2	-.0670
23.000	2	-.0642
23.250	2	-.0617
23.500	2	-.0596
23.750	2	-.0577
24.000	2	-.0560
24.250	2	-.0544

24.500	2	-.0530
24.750	2	-.0516
25.000	2	-.0502
25.250	2	-.0488
25.500	2	-.0473
25.750	2	-.0458
26.000	2	-.0442
26.250	2	-.0427
26.500	2	-.0411
26.750	2	-.0394
27.000	2	-.0378
27.250	2	-.0362
27.500	2	-.0345
27.750	2	-.0329
28.000	2	-.0312
28.250	2	-.0296
28.500	2	-.0280
28.750	2	-.0264
29.000	2	-.0248
29.250	2	-.0233
29.500	2	-.0218
29.750	2	-.0204
30.000	2	-.0190
5.250	3	-1296.4319
5.500	3	-810.5033
5.750	3	-501.4089
6.000	3	-305.4537
6.250	3	-182.8931
6.500	3	-107.5906
6.750	3	-62.3177
7.000	3	-35.8000
7.250	3	-20.8165
7.500	3	-12.6945
7.750	3	-8.5713
8.000	3	-6.6083
8.250	3	-5.7542
8.500	3	-5.4122
8.750	3	-5.3650
9.000	3	-5.3076
9.250	3	-5.1685
9.500	3	-4.9961
9.750	3	-4.7468
10.000	3	-4.4091
10.250	3	-4.0624
10.500	3	-3.7223
10.750	3	-3.3845
11.000	3	-3.0542
11.250	3	-2.7384

11.500	3	-2.4424
11.750	3	-2.1711
12.000	3	-1.9293
12.250	3	-1.7198
12.500	3	-1.5373
12.750	3	-1.3743
13.000	3	-1.2235
13.250	3	-1.0795
13.500	3	-.9445
13.750	3	-.8227
14.000	3	-.7184
14.250	3	-.6343
14.500	3	-.5678
14.750	3	-.5146
15.000	3	-.4706
15.250	3	-.4323
15.500	3	-.3985
15.750	3	-.3688
16.000	3	-.3427
16.250	3	-.3197
16.500	3	-.2992
16.750	3	-.2808
17.000	3	-.2640
17.250	3	-.2482
17.500	3	-.2331
17.750	3	-.2182
18.000	3	-.2034
18.250	3	-.1889
18.500	3	-.1747
18.750	3	-.1609
19.000	3	-.1475
19.250	3	-.1348
19.500	3	-.1226
19.750	3	-.1112
20.000	3	-.1005
20.250	3	-.0907
20.500	3	-.0817
20.750	3	-.0735
21.000	3	-.0660
21.250	3	-.0592
21.500	3	-.0531
21.750	3	-.0476
22.000	3	-.0427
22.250	3	-.0383
22.500	3	-.0344
22.750	3	-.0310
23.000	3	-.0279
23.250	3	-.0253

23.500	3	-.0230
23.750	3	-.0211
24.000	3	-.0193
24.250	3	-.0178
24.500	3	-.0165
24.750	3	-.0154
25.000	3	-.0143
25.250	3	-.0133
25.500	3	-.0124
25.750	3	-.0115
26.000	3	-.0107
26.250	3	-.0099
26.500	3	-.0092
26.750	3	-.0085
27.000	3	-.0079
27.250	3	-.0073
27.500	3	-.0068
27.750	3	-.0063
28.000	3	-.0059
28.250	3	-.0054
28.500	3	-.0050
28.750	3	-.0047
29.000	3	-.0043
29.250	3	-.0040
29.500	3	-.0037
29.750	3	-.0034
30.000	3	-.0032
5.250	4	2251.6612
5.500	4	1487.3122
5.750	4	979.0631
6.000	4	640.5275
6.250	4	416.0887
6.500	4	268.0061
6.750	4	170.9411
7.000	4	107.7834
7.250	4	67.0534
7.500	4	41.0515
7.750	4	24.6192
8.000	4	14.3100
8.250	4	7.9871
8.500	4	4.0940
8.750	4	1.8745
9.000	4	.5262
9.250	4	-.1919
9.500	4	-.4752
9.750	4	-.6565
10.000	4	-.6727
10.250	4	-.6336

10.500	4	-.5692
10.750	4	-.4756
11.000	4	-.3808
11.250	4	-.3132
11.500	4	-.2699
11.750	4	-.2405
12.000	4	-.2143
12.250	4	-.1833
12.500	4	-.1496
12.750	4	-.1178
13.000	4	-.0925
13.250	4	-.0771
13.500	4	-.0702
13.750	4	-.0694
14.000	4	-.0721
14.250	4	-.0759
14.500	4	-.0796
14.750	4	-.0822
15.000	4	-.0824
15.250	4	-.0796
15.500	4	-.0741
15.750	4	-.0663
16.000	4	-.0568
16.250	4	-.0461
16.500	4	-.0348
16.750	4	-.0235
17.000	4	-.0127
17.250	4	-.0029
17.500	4	.0053
17.750	4	.0115
18.000	4	.0158
18.250	4	.0185
18.500	4	.0198
18.750	4	.0198
19.000	4	.0190
19.250	4	.0174
19.500	4	.0153
19.750	4	.0130
20.000	4	.0107
20.250	4	.0086
20.500	4	.0067
20.750	4	.0049
21.000	4	.0034
21.250	4	.0021
21.500	4	.0009
21.750	4	-.0001
22.000	4	-.0009
22.250	4	-.0016

22.500	4	-.0022
22.750	4	-.0026
23.000	4	-.0030
23.250	4	-.0032
23.500	4	-.0034
23.750	4	-.0035
24.000	4	-.0035
24.250	4	-.0035
24.500	4	-.0034
24.750	4	-.0034
25.000	4	-.0032
25.250	4	-.0031
25.500	4	-.0030
25.750	4	-.0028
26.000	4	-.0027
26.250	4	-.0025
26.500	4	-.0024
26.750	4	-.0022
27.000	4	-.0021
27.250	4	-.0019
27.500	4	-.0017
27.750	4	-.0015
28.000	4	-.0014
28.250	4	-.0012
28.500	4	-.0010
28.750	4	-.0008
29.000	4	-.0006
29.250	4	-.0005
29.500	4	-.0003
29.750	4	-.0001
30.000	4	.0001
5.250	5	-1034.5417
5.500	5	-663.5585
5.750	5	-427.1985
6.000	5	-273.9745
6.250	5	-174.6811
6.500	5	-110.4919
6.750	5	-69.1500
7.000	5	-42.7718
7.250	5	-26.1563
7.500	5	-15.7528
7.750	5	-9.3828
8.000	5	-5.5109
8.250	5	-3.2281
8.500	5	-1.7367
8.750	5	-1.0432
9.000	5	-.5780
9.250	5	-.3488

9.500	5	-.1556
9.750	5	-.1563
10.000	5	-.1034
10.250	5	-.0971
10.500	5	-.1331
10.750	5	-.1559
11.000	5	-.1580
11.250	5	-.1442
11.500	5	-.1199
11.750	5	-.0910
12.000	5	-.0631
12.250	5	-.0411
12.500	5	-.0253
12.750	5	-.0153
13.000	5	-.0105
13.250	5	-.0100
13.500	5	-.0109
13.750	5	-.0102
14.000	5	-.0046
14.250	5	.0081
14.500	5	.0256
14.750	5	.0448
15.000	5	.0625
15.250	5	.0762
15.500	5	.0858
15.750	5	.0916
16.000	5	.0943
16.250	5	.0942
16.500	5	.0920
16.750	5	.0881
17.000	5	.0829
17.250	5	.0771
17.500	5	.0710
17.750	5	.0651
18.000	5	.0595
18.250	5	.0542
18.500	5	.0491
18.750	5	.0443
19.000	5	.0399
19.250	5	.0357
19.500	5	.0319
19.750	5	.0283
20.000	5	.0251
20.250	5	.0222
20.500	5	.0195
20.750	5	.0172
21.000	5	.0151
21.250	5	.0133

21.500	5	.0117
21.750	5	.0104
22.000	5	.0092
22.250	5	.0082
22.500	5	.0074
22.750	5	.0067
23.000	5	.0061
23.250	5	.0057
23.500	5	.0054
23.750	5	.0051
24.000	5	.0049
24.250	5	.0047
24.500	5	.0046
24.750	5	.0044
25.000	5	.0043
25.250	5	.0041
25.500	5	.0039
25.750	5	.0037
26.000	5	.0034
26.250	5	.0032
26.500	5	.0029
26.750	5	.0026
27.000	5	.0023
27.250	5	.0020
27.500	5	.0016
27.750	5	.0013
28.000	5	.0009
28.250	5	.0006
28.500	5	.0002
28.750	5	-.0001
29.000	5	-.0005
29.250	5	-.0008
29.500	5	-.0012
29.750	5	-.0015
30.000	5	-.0018
5.250	6	897.5629
5.500	6	567.1025
5.750	6	362.4928
6.000	6	232.3053
6.250	6	148.9237
6.500	6	95.3231
6.750	6	60.7329
7.000	6	38.4798
7.250	6	24.1870
7.500	6	15.1106
7.750	6	9.3120
8.000	6	5.6937
8.250	6	3.4827

8.500	6	1.9272
8.750	6	1.1759
9.000	6	.6747
9.250	6	.3806
9.500	6	.1543
9.750	6	.1257
10.000	6	.0598
10.250	6	-.0005
10.500	6	-.0283
10.750	6	-.0321
11.000	6	-.0304
11.250	6	-.0396
11.500	6	-.0588
11.750	6	-.0825
12.000	6	-.1055
12.250	6	-.1229
12.500	6	-.1332
12.750	6	-.1352
13.000	6	-.1282
13.250	6	-.1119
13.500	6	-.0897
13.750	6	-.0659
14.000	6	-.0446
14.250	6	-.0292
14.500	6	-.0196
14.750	6	-.0149
15.000	6	-.0142
15.250	6	-.0166
15.500	6	-.0215
15.750	6	-.0284
16.000	6	-.0367
16.250	6	-.0459
16.500	6	-.0553
16.750	6	-.0645
17.000	6	-.0729
17.250	6	-.0798
17.500	6	-.0849
17.750	6	-.0876
18.000	6	-.0882
18.250	6	-.0869
18.500	6	-.0840
18.750	6	-.0798
19.000	6	-.0746
19.250	6	-.0688
19.500	6	-.0625
19.750	6	-.0562
20.000	6	-.0500
20.250	6	-.0444

20.500	6	-.0392
20.750	6	-.0345
21.000	6	-.0302
21.250	6	-.0263
21.500	6	-.0228
21.750	6	-.0197
22.000	6	-.0170
22.250	6	-.0145
22.500	6	-.0124
22.750	6	-.0105
23.000	6	-.0089
23.250	6	-.0075
23.500	6	-.0063
23.750	6	-.0052
24.000	6	-.0044
24.250	6	-.0037
24.500	6	-.0030
24.750	6	-.0025
25.000	6	-.0020
25.250	6	-.0016
25.500	6	-.0012
25.750	6	-.0008
26.000	6	-.0005
26.250	6	-.0002
26.500	6	.0001
26.750	6	.0003
27.000	6	.0005
27.250	6	.0007
27.500	6	.0009
27.750	6	.0010
28.000	6	.0012
28.250	6	.0013
28.500	6	.0014
28.750	6	.0015
29.000	6	.0015
29.250	6	.0016
29.500	6	.0016
29.750	6	.0017
30.000	6	.0017
5.250	7	-481.1059
5.500	7	-291.6166
5.750	7	-181.6207
6.000	7	-114.2237
6.250	7	-72.3330
6.500	7	-45.8880
6.750	7	-28.9961
7.000	7	-18.2370
7.250	7	-11.3893

7.500	7	-7.0513
7.750	7	-4.3241
8.000	7	-2.6190
8.250	7	-1.6044
8.500	7	-.8370
8.750	7	-.5997
9.000	7	-.3260
9.250	7	-.2108
9.500	7	-.2257
9.750	7	-.0971
10.000	7	-.1043
10.250	7	-.0855
10.500	7	-.0298
10.750	7	.0056
11.000	7	.0238
11.250	7	.0402
11.500	7	.0566
11.750	7	.0716
12.000	7	.0839
12.250	7	.0923
12.500	7	.0963
12.750	7	.0957
13.000	7	.0902
13.250	7	.0796
13.500	7	.0644
13.750	7	.0450
14.000	7	.0218
14.250	7	-.0041
14.500	7	-.0303
14.750	7	-.0538
15.000	7	-.0716
15.250	7	-.0813
15.500	7	-.0836
15.750	7	-.0795
16.000	7	-.0705
16.250	7	-.0575
16.500	7	-.0420
16.750	7	-.0250
17.000	7	-.0078
17.250	7	.0084
17.500	7	.0224
17.750	7	.0333
18.000	7	.0411
18.250	7	.0462
18.500	7	.0489
18.750	7	.0498
19.000	7	.0491
19.250	7	.0471

19.500	7	.0444
19.750	7	.0412
20.000	7	.0379
20.250	7	.0348
20.500	7	.0320
20.750	7	.0295
21.000	7	.0271
21.250	7	.0250
21.500	7	.0232
21.750	7	.0215
22.000	7	.0200
22.250	7	.0186
22.500	7	.0174
22.750	7	.0164
23.000	7	.0154
23.250	7	.0146
23.500	7	.0138
23.750	7	.0132
24.000	7	.0126
24.250	7	.0120
24.500	7	.0115
24.750	7	.0109
25.000	7	.0104
25.250	7	.0099
25.500	7	.0093
25.750	7	.0088
26.000	7	.0082
26.250	7	.0076
26.500	7	.0071
26.750	7	.0065
27.000	7	.0059
27.250	7	.0053
27.500	7	.0048
27.750	7	.0042
28.000	7	.0036
28.250	7	.0031
28.500	7	.0025
28.750	7	.0020
29.000	7	.0015
29.250	7	.0010
29.500	7	.0005
29.750	7	.0000
30.000	7	-.0004
5.250	8	346.6572
5.500	8	197.3724
5.750	8	117.5926
6.000	8	71.2361
6.250	8	43.8827

6.500	8	27.3108
6.750	8	17.0492
7.000	8	10.6117
7.250	8	6.6534
7.500	8	4.1150
7.750	8	2.5431
8.000	8	1.5754
8.250	8	.9359
8.500	8	.3689
8.750	8	.3550
9.000	8	.2114
9.250	8	.1481
9.500	8	.1821
9.750	8	.1752
10.000	8	.1258
10.250	8	.1159
10.500	8	.1190
10.750	8	.0910
11.000	8	.0491
11.250	8	.0198
11.500	8	.0055
11.750	8	.0027
12.000	8	.0080
12.250	8	.0179
12.500	8	.0291
12.750	8	.0384
13.000	8	.0426
13.250	8	.0397
13.500	8	.0326
13.750	8	.0253
14.000	8	.0219
14.250	8	.0255
14.500	8	.0343
14.750	8	.0455
15.000	8	.0562
15.250	8	.0641
15.500	8	.0690
15.750	8	.0713
16.000	8	.0715
16.250	8	.0698
16.500	8	.0666
16.750	8	.0624
17.000	8	.0575
17.250	8	.0522
17.500	8	.0471
17.750	8	.0422
18.000	8	.0378
18.250	8	.0338

18.500	8	.0301
18.750	8	.0268
19.000	8	.0237
19.250	8	.0209
19.500	8	.0183
19.750	8	.0159
20.000	8	.0137
20.250	8	.0116
20.500	8	.0096
20.750	8	.0078
21.000	8	.0061
21.250	8	.0046
21.500	8	.0031
21.750	8	.0018
22.000	8	.0006
22.250	8	-.0005
22.500	8	-.0015
22.750	8	-.0023
23.000	8	-.0031
23.250	8	-.0038
23.500	8	-.0044
23.750	8	-.0049
24.000	8	-.0053
24.250	8	-.0056
24.500	8	-.0059
24.750	8	-.0060
25.000	8	-.0061
25.250	8	-.0061
25.500	8	-.0061
25.750	8	-.0060
26.000	8	-.0058
26.250	8	-.0056
26.500	8	-.0054
26.750	8	-.0051
27.000	8	-.0047
27.250	8	-.0043
27.500	8	-.0039
27.750	8	-.0035
28.000	8	-.0030
28.250	8	-.0025
28.500	8	-.0021
28.750	8	-.0016
29.000	8	-.0011
29.250	8	-.0006
29.500	8	-.0001
29.750	8	.0004
30.000	8	.0009
5.250	9	-217.7613

5.500	9	-114.1183
5.750	9	-64.3094
6.000	9	-37.1029
6.250	9	-22.0121
6.500	9	-13.4215
6.750	9	-8.2992
7.000	9	-5.1637
7.250	9	-3.1938
7.500	9	-1.9961
7.750	9	-1.2748
8.000	9	-.7849
8.250	9	-.4571
8.500	9	-.0027
8.750	9	-.1529
9.000	9	-.1414
9.250	9	-.1172
9.500	9	-.0641
9.750	9	-.1377
10.000	9	-.1409
10.250	9	-.1195
10.500	9	-.1093
10.750	9	-.1136
11.000	9	-.1204
11.250	9	-.1175
11.500	9	-.1081
11.750	9	-.0988
12.000	9	-.0964
12.250	9	-.1055
12.500	9	-.1207
12.750	9	-.1347
13.000	9	-.1400
13.250	9	-.1309
13.500	9	-.1101
13.750	9	-.0822
14.000	9	-.0515
14.250	9	-.0223
14.500	9	.0037
14.750	9	.0249
15.000	9	.0401
15.250	9	.0482
15.500	9	.0498
15.750	9	.0461
16.000	9	.0380
16.250	9	.0267
16.500	9	.0131
16.750	9	-.0017
17.000	9	-.0167
17.250	9	-.0308

17.500	9	-.0430
17.750	9	-.0523
18.000	9	-.0591
18.250	9	-.0634
18.500	9	-.0656
18.750	9	-.0661
19.000	9	-.0651
19.250	9	-.0630
19.500	9	-.0599
19.750	9	-.0563
20.000	9	-.0525
20.250	9	-.0486
20.500	9	-.0448
20.750	9	-.0410
21.000	9	-.0373
21.250	9	-.0337
21.500	9	-.0302
21.750	9	-.0268
22.000	9	-.0234
22.250	9	-.0202
22.500	9	-.0172
22.750	9	-.0142
23.000	9	-.0114
23.250	9	-.0088
23.500	9	-.0063
23.750	9	-.0040
24.000	9	-.0019
24.250	9	.0000
24.500	9	.0017
24.750	9	.0032
25.000	9	.0044
25.250	9	.0055
25.500	9	.0063
25.750	9	.0069
26.000	9	.0073
26.250	9	.0075
26.500	9	.0076
26.750	9	.0075
27.000	9	.0072
27.250	9	.0069
27.500	9	.0064
27.750	9	.0058
28.000	9	.0052
28.250	9	.0044
28.500	9	.0036
28.750	9	.0028
29.000	9	.0019
29.250	9	.0011

29.500	9	.0002
29.750	9	-.0007
30.000	9	-.0016
30.250	9	-.0024
30.500	9	-.0032
30.750	9	-.0039
31.000	9	-.0046
31.250	9	-.0053
31.500	9	-.0060
31.750	9	-.0066
32.000	9	-.0072
32.250	9	-.0077
32.500	9	-.0082
32.750	9	-.0087
33.000	9	-.0092
5.250	10	197.7597
5.500	10	91.5781
5.750	10	46.9772
6.000	10	24.5980
6.250	10	13.4385
6.500	10	7.6987
6.750	10	4.5270
7.000	10	2.7657
7.250	10	1.7013
7.500	10	1.0356
7.750	10	.6836
8.000	10	.4106
8.250	10	.2545
8.500	10	-.2526
8.750	10	.1040
9.000	10	.0554
9.250	10	.0281
9.500	10	.0107
9.750	10	-.0216
10.000	10	-.0192
10.250	10	-.0031
10.500	10	.0142
10.750	10	.0287
11.000	10	.0401
11.250	10	.0491
11.500	10	.0565
11.750	10	.0636
12.000	10	.0712
12.250	10	.0800
12.500	10	.0881
12.750	10	.0929
13.000	10	.0921
13.250	10	.0838

13.500	10	.0689
13.750	10	.0489
14.000	10	.0254
14.250	10	.0001
14.500	10	-.0249
14.750	10	-.0471
15.000	10	-.0640
15.250	10	-.0739
15.500	10	-.0772
15.750	10	-.0749
16.000	10	-.0681
16.250	10	-.0577
16.500	10	-.0449
16.750	10	-.0307
17.000	10	-.0161
17.250	10	-.0022
17.500	10	.0101
17.750	10	.0199
18.000	10	.0272
18.250	10	.0323
18.500	10	.0356
18.750	10	.0371
19.000	10	.0374
19.250	10	.0365
19.500	10	.0348
19.750	10	.0325
20.000	10	.0299
20.250	10	.0273
20.500	10	.0246
20.750	10	.0219
21.000	10	.0193
21.250	10	.0166
21.500	10	.0140
21.750	10	.0114
22.000	10	.0089
22.250	10	.0064
22.500	10	.0041
22.750	10	.0018
23.000	10	-.0004
23.250	10	-.0025
23.500	10	-.0045
23.750	10	-.0063
24.000	10	-.0080
24.250	10	-.0095
24.500	10	-.0109
24.750	10	-.0120
25.000	10	-.0130
25.250	10	-.0138

25.500	10	-.0144
25.750	10	-.0148
26.000	10	-.0150
26.250	10	-.0151
26.500	10	-.0151
26.750	10	-.0149
27.000	10	-.0146
27.250	10	-.0141
27.500	10	-.0136
27.750	10	-.0130
28.000	10	-.0124
28.250	10	-.0116
28.500	10	-.0108
28.750	10	-.0100
29.000	10	-.0092
29.250	10	-.0083
29.500	10	-.0074
29.750	10	-.0066
30.000	10	-.0058
5.250	11	-203.2202
5.500	11	-85.6728
5.750	11	-40.9156
6.000	11	-19.5549
6.250	11	-9.7463
6.500	11	-5.1627
6.750	11	-2.8603
7.000	11	-1.6512
7.250	11	-1.0504
7.500	11	-.6390
7.750	11	-.3516
8.000	11	-.1833
8.250	11	-.1405
8.500	11	.4761
8.750	11	-.0762
9.000	11	-.0322
9.250	11	.0118
9.500	11	.0672
9.750	11	.1284
10.000	11	.1311
10.250	11	.1176
10.500	11	.1102
10.750	11	.1148
11.000	11	.1178
11.250	11	.1054
11.500	11	.0823
11.750	11	.0577
12.000	11	.0411
12.250	11	.0389

12.500	11	.0470
12.750	11	.0584
13.000	11	.0662
13.250	11	.0650
13.500	11	.0561
13.750	11	.0428
14.000	11	.0279
14.250	11	.0141
14.500	11	.0023
14.750	11	-.0073
15.000	11	-.0143
15.250	11	-.0184
15.500	11	-.0199
15.750	11	-.0191
16.000	11	-.0166
16.250	11	-.0126
16.500	11	-.0076
16.750	11	-.0021
17.000	11	.0037
17.250	11	.0093
17.500	11	.0142
17.750	11	.0182
18.000	11	.0213
18.250	11	.0236
18.500	11	.0252
18.750	11	.0262
19.000	11	.0267
19.250	11	.0268
19.500	11	.0267
19.750	11	.0263
20.000	11	.0258
20.250	11	.0253
20.500	11	.0249
20.750	11	.0244
21.000	11	.0240
21.250	11	.0235
21.500	11	.0231
21.750	11	.0226
22.000	11	.0222
22.250	11	.0217
22.500	11	.0213
22.750	11	.0208
23.000	11	.0204
23.250	11	.0199
23.500	11	.0194
23.750	11	.0189
24.000	11	.0184
24.250	11	.0179

24.500	11	.0173
24.750	11	.0167
25.000	11	.0161
25.250	11	.0155
25.500	11	.0149
25.750	11	.0142
26.000	11	.0135
26.250	11	.0128
26.500	11	.0121
26.750	11	.0114
27.000	11	.0106
27.250	11	.0099
27.500	11	.0091
27.750	11	.0084
28.000	11	.0076
28.250	11	.0069
28.500	11	.0062
28.750	11	.0055
29.000	11	.0047
29.250	11	.0040
29.500	11	.0034
29.750	11	.0027
30.000	11	.0021
5.250	12	99.2122
5.500	12	40.6278
5.750	12	18.9406
6.000	12	8.7644
6.250	12	4.1887
6.500	12	2.1331
6.750	12	1.1538
7.000	12	.6411
7.250	12	.4089
7.500	12	.2443
7.750	12	.1319
8.000	12	.0491
8.250	12	.0543
8.500	12	-.2667
8.750	12	.0246
9.000	12	.0297
9.250	12	.0078
9.500	12	-.0181
9.750	12	-.0426
10.000	12	-.0547
10.250	12	-.0747
10.500	12	-.0939
10.750	12	-.0986
11.000	12	-.0909
11.250	12	-.0769

11.500	12	-.0612
11.750	12	-.0481
12.000	12	-.0417
12.250	12	-.0450
12.500	12	-.0541
12.750	12	-.0638
13.000	12	-.0690
13.250	12	-.0655
13.500	12	-.0550
13.750	12	-.0400
14.000	12	-.0232
14.250	12	-.0072
14.500	12	.0071
14.750	12	.0191
15.000	12	.0280
15.250	12	.0333
15.500	12	.0353
15.750	12	.0345
16.000	12	.0315
16.250	12	.0266
16.500	12	.0205
16.750	12	.0136
17.000	12	.0064
17.250	12	-.0006
17.500	12	-.0069
17.750	12	-.0121
18.000	12	-.0162
18.250	12	-.0193
18.500	12	-.0216
18.750	12	-.0231
19.000	12	-.0241
19.250	12	-.0244
19.500	12	-.0244
19.750	12	-.0241
20.000	12	-.0237
20.250	12	-.0231
20.500	12	-.0225
20.750	12	-.0219
21.000	12	-.0212
21.250	12	-.0204
21.500	12	-.0196
21.750	12	-.0188
22.000	12	-.0180
22.250	12	-.0171
22.500	12	-.0163
22.750	12	-.0154
23.000	12	-.0145
23.250	12	-.0136

23.500	12	-.0127
23.750	12	-.0118
24.000	12	-.0110
24.250	12	-.0101
24.500	12	-.0093
24.750	12	-.0085
25.000	12	-.0077
25.250	12	-.0070
25.500	12	-.0063
25.750	12	-.0057
26.000	12	-.0051
26.250	12	-.0045
26.500	12	-.0039
26.750	12	-.0034
27.000	12	-.0029
27.250	12	-.0025
27.500	12	-.0020
27.750	12	-.0016
28.000	12	-.0012
28.250	12	-.0008
28.500	12	-.0005
28.750	12	-.0001
29.000	12	.0002
29.250	12	.0005
29.500	12	.0008
29.750	12	.0011
30.000	12	.0013
